# Analysis of intention and influencing factors on mobile information follow-up service in HIV/AIDS in a city in China

**DOI:** 10.3389/fpubh.2022.997681

**Published:** 2022-11-10

**Authors:** Chuancang Li, Pengli Wang, Mengge Zhang, Mengbing Qu, Qian Cai, Jingjing Meng, Haohao Fan, Liang Sun

**Affiliations:** ^1^Department of Social Medicine and Health Service Management, College of Public Health, Zhengzhou University, Zhengzhou, China; ^2^Shanghai Qingpu District Center for Disease Control and Prevention, Shanghai, China

**Keywords:** mobile follow-up service, technology acceptance model, AIDS, willingness to behave, structural equation model

## Abstract

**Objectives:**

This study aimed to evaluate the willingness of patients with HIV/AIDS in Henan province to accept mobile information follow-up, to find the key factors that affect behavioral willingness to accept such follow-up, to explore the internal mechanism of the mobile service, and to provide a theoretical rationale for the further promotion of mobile follow-up.

**Methods:**

This study used the technology acceptance model (TAM) as its main theoretical tool, which adopted a stratified random sampling method, and investigated 284 patients with HIV/AIDS in area six of Sanmenxia City. An on-site questionnaire survey method was adopted for this study. Confirmatory factor analysis was used for structural validity, with Cronbach's coefficient used for reliability. Data analysis mainly used SPSS23.0 and AMOS23.0 software.

**Results:**

The acceptance rate of the HIV/AIDS mobile follow-up service was 68.53%. In the study, product factors (PFs) were considered important in the indirect path of the TAM. Our TAM model suggested that high perceived usefulness (PU), perceived ease of use (PEU), and perceived innovativeness (PI) of the service were significant in improving mobile health (mHealth) acceptance among patients with HIV/AIDS in China. Subjective norms (SNs) also contributed to popularizing the service in the HIV/AIDS community. The model fitting was considered acceptable (root mean square error of approximation, RMSEA = 0.074; goodness of fit index, GFI = 0.905; comparative fit index, CFI = 0.963, and Tucker-Lewis index, TLI = 0.593).

**Conclusion:**

PFs and SNs exerted an important influence on the behavioral intentions of the patients with HIV/AIDS who accepted mobile health. PU was another important factor affecting behavioral intention. The practicality of mHealth services was crucial. Convenience and the innovativeness of the experience with the service will be conducive to the promotion and use of mHealth follow-up services.

## Introduction

According to the WHO data, in 2020 (1), about 37.7 million people around the world were infected with HIV, 1.5 million of whom were newly infected, while 680,000 people died from HIV-related causes. A large number of HIV-infected people raises many issues, such as providing safe and appropriate care for them. In recent years, due to the implementation of measures, such as the expansion in the scope of testing and free treatment, follow-up services, which were solely reliant on the work of the Centers for Disease Control and Prevention (CDC), were overwhelmed. In addition, some patients with HIV/AIDS feared that their privacy would be compromised, potentially exposing them to social discrimination and stigma. These two reasons may have hindered their inclination to obtain follow-up care (2, 3). Although there were some interventions in place to support patients with HIV/AIDS patients in China, many were missing out on the follow-up. It remained a serious problem (4, 5).

Mobile technology has become increasingly pervasive in health care (6), enabling mobile health (mHealth) to emerge as a valuable healthcare model, a model based on mobile communication, network technology, and special tool technology. It uses a combination of electronic health and smartphone technology (7), providing health services and health information (8, 9) through mobile communication technology (personal digital assistants, mobile phones, satellite communication, and wearable sensors). mHealth services rely on mobile information technology platforms to have cross-regional and cross-time advantages. They can cover a large number of users in a short period, and are low-cost, highly targeted, convenient, and fast. While they have been used in managing other diseases (10, 11), as time passed, the use of medical and health applications gradually declined among those patients, such that after 90 days only about 27 and 30% of patients, respectively (12), were still using the applications. Therefore, there is a need to conduct a thorough examination of the user needs to guide the development of new health-related applications and to ensure that the content continues to be attractive to them over time.

In 1989, Davis proposed the technology acceptance model (TAM) to study the degree of acceptance of emerging technologies (13). The TAM points out that the intention to use a certain technology is affected by the attitude of the user toward its use, subjective norms (SNs), perceived usefulness (PU), ease of use, and by other related variables (14). These factors can be used to explain why users accept or reject an information system. The TAM developed from these premises can predict and estimate people's acceptance of a certain emerging technology. Since the TAM was proposed, many researchers have modified it to improve its interpretability to create a model with higher predictive capabilities (15–17), and, given the characteristics of mHealth products, in this study, we have added perceived innovativeness factors to further enhance the model. This study used the improved TAM model to examine the behavioral intentions of patients with HIV/AIDS receiving mobile follow-up services, to explore the factors that affect patients with HIV/AIDS in deciding whether to receive mobile follow-up, and to provide a theoretical rationale to support mobile follow-up.

## Study population and methods

### Study population

The study population included all patients with HIV/AIDS registered in the CDC of Sanmenxia City, Henan Province, as of 2018. Inclusion criteria were as follows: (1) sober consciousness, normal intelligence, and no previous history of mental illness; (2) no literacy impairment; (3) showing clarity about the purpose of this research and volunteering to participate in the survey. Exclusion criteria included the following: (1) severe neuropsychological disorder or mental illness, without the capacity for autonomous judgment and (2) inability to communicate effectively.

### Sample

According to the international scale design guidelines, the sample size calculation method of this questionnaire survey refers to the experience of multivariate analysis. The ratio of the sample size to the estimated parameters should be at least 5:1, and, if possible, it should be as high as 10:1 as a reference (18). Considering the potential for losses to a follow-up of 5–10% of cases, the estimated sample size was 179.

In this study, a stratified random sampling method was adopted. Based on the estimated sample size and the number of patients with HIV/AIDS in each area, patients were randomly selected in area six of Sanmenxia City from among those registered in the Sanmenxia Disease Control System. First, HIV-infected people in the 6 districts and counties of Sanmenxia City were divided into high, middle, and low strata with two districts and counties in each stratum, and then patients were randomly sampled by proportion from each stratum to participate in this survey.

### Questionnaire and data collection

The self-report questionnaire used in this survey consisted of two parts. The first part mostly covered basic personal information of patients, including their demographic characteristics and disease conditions. The second part included 6 factors and 17 questions in total (Supplementary Tables). There were 4 questions about product factors (PFs), 2 questions about SNs, 3 questions about perceived ease of use (PEU), 3 questions about PU, and 2 questions about perceived innovativeness of the service (PI). To test potential outcomes, three questions involved the behavioral intentions (BI) of patients, that is, their intention to accept or refuse HIV/AIDS mobile information follow-up.

The numbers corresponding to the options in this section reflect the degree of agreement of the respondent with each question, that is, their subjective evaluation of the issue in the question. The measurement index was a Likert 5-point scale. The degree of agreement increases from 1 to 5. The higher scores indicate more support from the respondent, while the lower scores indicate more opposition. A score of 1 indicated strong disagreement with the statement in the question, while a score of 5 indicated strong agreement with the corresponding statement. The content and structure of the questionnaire were both approved by a panel of experts. For the reliability and validity evaluation of the scale, the overall Cronbach's alpha coefficient was 0.963, the KMO was 0.949, the value of Bartlett's sphere test was 4,492.046, *p* < 0.05, and the four factors cumulatively explained 78.85% of the total variance.

An on-site self-report questionnaire survey method was adopted in this study. After researchers consulted with the staff of the local CDC, respondents were collectively called in for their regular HIV/AIDS physical examination, after which researchers clarified the purpose and significance of the investigation before distributing the questionnaire. The survey subjects were asked to fill out the questionnaire independently, and not to communicate with others. They were informed that there was no correct answer to any question and that they needed to answer truthfully, in accordance with their real-life situation.

### Statistical analysis

Epidata3.2 software was used to input data, and SPSS23.0 software was used for statistical analysis. Continuous variables were described by mean ± standard deviation, while categorical variables were described by frequency and percentage. AMOS23.0 software was used for structure equation model (SEM) data fitting. All analyses were two-tailed, and a *p*-value of <0.05 was considered statistically significant.

### Model evaluation indicators

χ^2^/*df* was used to evaluate the difference between the sample and the estimated model. The χ^2^/*df* between 1 and 3 indicated a good model fit. Root mean square error of approximation (RMSEA) was the most commonly used fitting index, with <0.08 considered a good fit, and 0.08–0.10 considered a general fit. The goodness of fit index (GFI) was between 0 and 1, equivalent to the coefficient *R*^2^ in regression analysis. The closer the value was to 1, the better the fit of the model. In general, the GFI value of >0.9 indicated that the model path graph was a good fit with the actual data. The standard root mean square residual (SRMR) was between 0 and 1, with the acceptable value of model fit being <0.05. Tucker-Lewis index (TLI) value of >0.9 was considered to be a good fit. The comparative fit index (CFI) was between 0 (the model does not fit at all) and 1 (the model fits completely). The CFI value of >0.9 indicated that the model path graph was a good fit with the actual data (19).

## Results

### Social demographic characteristics of HIV/AIDS

The respondents were 237 men (83.45%) and 47 women (16.55%), who were under 35 years old mostly, accounting for 36.27% of all respondents. The largest occupation group was farmers of about 128 people, accounting for 45.07% of respondents, followed by workers of about 55 (19.37%), while civil servants/cadres comprised the smallest group with only 5 people (1.76%). Among the respondents, 149 were patients with AIDS (52.46%) and 135 were people with asymptomatic HIV infections (47.54%). The behavioral intention score of patients with HIV/AIDS was 10.28 ± 4.31, with 68.53% of respondents indicating acceptance of the mobile follow-up. All results are shown in Table 1.

**Table 1:** Characteristics of participants (*n* = 284).

**Characteristics**	**Respondent (*n*)**	**Percentage (%)**
**Gender**		
Male	237	83.45
Female	47	16.55
**Age group**		
<35	103	36.27
35~	83	29.23
45~	52	18.31
55~	30	10.56
65~	16	5.63
**Educational level**		
Primary school and below	33	11.62
Middle school	193	67.96
College and above	58	20.42
**Occupation**		
Farmer	128	45.07
Workers (migrant workers)	55	19.37
Self-employed	22	7.75
Student	8	2.82
Civil servants/cadres	5	1.76
Retirees	7	2.46
Others	59	20.77
Average income^*^		
<1000	145	51.06
1000~	105	36.97
3000~	28	9.86
5000~	4	1.41
**Marital status**		
Getting married or cohabiting	147	51.76
Single	137	48.24
**Disease status**		
HIV infection	135	47.54
AIDS patient	149	52.46

* Missing two people.

### Correlation analysis and regression analysis between model variables

With demographic characteristics as control variables, the Pearson correlation coefficients of each factor, including PFs, SNs, PEU, PU, and PI with BI, were, respectively, 0.705, 0.717, 0.727, 0.782, and 0.778. All factors were significantly associated with BI. The results are shown in Table 2.

**Table 2:** The correlation between behavioral intention and other constructs.

**Construct**	**Pearson** **(*r*)**	***P*** **-value**	**Regression** **(*R*^2^)**	***P*** **-value**
Product factors	0.705	<0.001	0.117	0.026
Subjective norms	0.717	<0.001	0.193	<0.001
Perceived ease of use	0.727	<0.001	0.126	0.038
Perceived usefulness	0.782	<0.001	0.267	<0.001
Perceived innovativeness	0.778	<0.001	0.268	<0.001

## SEM analysis of influencing factors in behavioral willingness to accept mobile information follow-up

### Construction of the initial model of mobile information follow-up behavioral willingness

Multiple linear regression analysis was used to analyze the effect path of each influencing factor and of behavioral intentions, all of which independently affected the behavioral intentions of participants, including three behavioral intentions which were incorporated into the structural equation model. The maximum likelihood method was used to continuously fit the initial model. Through comprehensive consideration of the correction index, standardized residuals, path coefficient *p*-values, and deletion of meaningless paths and variables, a modified model with an ideal fitting effect was finally obtained. The result of the model path coefficient estimation is shown in Table 3.

**Table 3:** Model path coefficient estimation.

**Relationship**			**PC**	**SPC**	**S.E**.	**C.R**.	***P*** **-value**
PF	→	PEU	1.154	0.889	0.058	19.726	<0.001
PF	→	PU	1.117	0.974	0.035	31.918	<0.001
PF	→	PI	1.151	0.912	0.069	16.652	<0.001
PF	→	BI 3	0.320	0.205	0.148	2.167	0.030
SN	→	BI 1	0.362	0.247	0.062	5.845	<0.001
SN	→	BI 2	0.706	0.463	0.076	9.228	<0.001
SN	→	BI 3	0.788	0.506	0.091	8.651	<0.001
PEU	→	PU	0.126	0.143	0.062	2.032	0.042
PEU	→	BI 2	0.281	0.240	0.072	3.931	<0.001
PU	→	BI 3	0.729	0.537	0.127	5.764	<0.001
PI	→	BI 1	0.959	0.824	0.054	17.808	<0.001
PI	→	BI 2	0.732	0.607	0.079	9.297	<0.001

PC, path coefficient; SPC, standardized path coefficient; PFs, product factors; SNs, subjective norms; PEU, perceived ease of use; PU, perceived usefulness; PI, perceived innovativeness; BI 1, behavioral intention1; BI 2, behavioral intention2; BI 3, behavioral intention 3.

**Figure 1:**
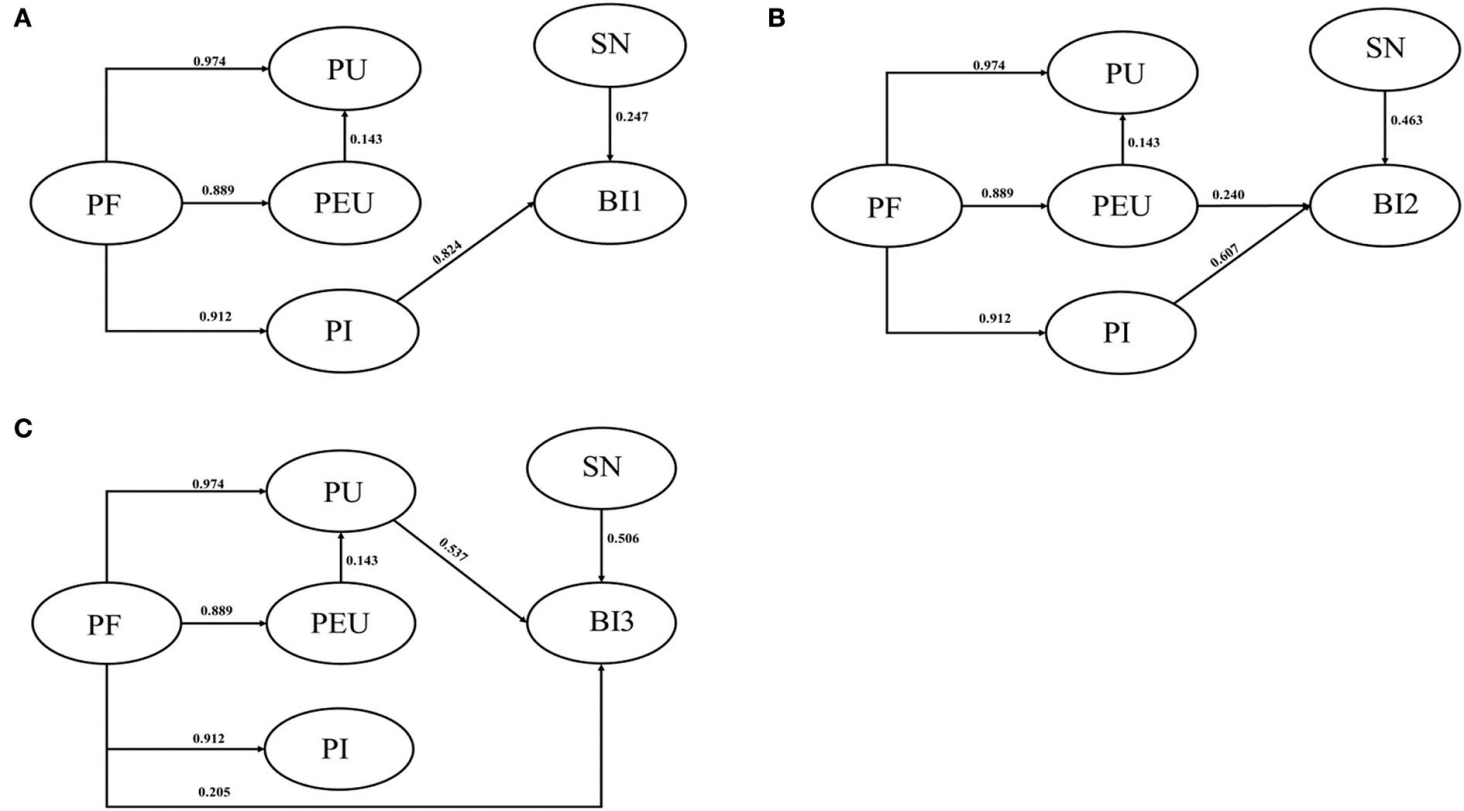
**(A)** Outcome with BI1; **(B)** outcome with BI2; **(C)** outcome with BI3. PF, product factor; PU, perceived usefulness; PEU, perceived ease of use; PI, perceived innovation; SN, subjective norm; BI, behavioral intention.

As shown in Figure 1, the effect relationship of each related variable in the model was analyzed. The effect relationship between the variables in the SEM diagram is presented in a quantitative form in which the direct effect is equivalent to the path coefficient, the indirect effect is equivalent to multiplying the corresponding path coefficients, and the addition of the direct and indirect effects is regarded as the total effect.

Product factors, SNs, and PI can all affect BI 1, the path coefficients being 0.751, 0.247, and 0.842, respectively. SNs and PI affected BI 1 directly, while PFs affected BI 1 indirectly. The indirect effect path was PFs → PI → BI 1. PFs, SNs, PEU, and PI all affected BI 2, with path coefficients of 0.767, 0.463, 0.240, and 0.607, respectively. Among them, SNs, PEU, and PI directly affected BI 2, while PFs indirectly affected it. There were two indirect effect paths: (1) PFs → PEU → BI 2, and (2) PFs → PI → BI 2. PFs, SNs, PEU, and PU all affected BI 3. Among them, PEU indirectly affected BI 3; the path coefficient was 0.077, and the specific path was as follows: PEU → PU → BI 3. SNs and PU directly affected BI 3, the path coefficients being 0.506 and 0.537, respectively. PFs affected BI 3 both directly and indirectly, and there were two indirect paths: (1) PFs → PEU → PU → BI 3, and (2) PFs → PU → BI 3.

### Model fitting effect evaluation

The analysis of our proposed TAM model suggested that the model met parameters based on the goodness of fit test. The model should therefore be considered acceptable. More details are shown in Table 4 below.

**Table 4:** Fitting index of the revised model.

**The goodness of fit indices**	**Cut-off value**	**Result**	**Status**
χ^2^/df	<3.00	2.546	Acceptable
Root mean square error of approximation (RMSEA)	<0.10	0.074	Acceptable
Goodness of fit index (GFI)	>0.90	0.905	Acceptable
Standard root mean square residual (SRMR)	<0.05	0.044	Acceptable
Tucker-Lewis index (TLI)	>0.90	0.953	Acceptable
Comparative fit index (CFI)	>0.90	0.963	Acceptable

## Discussion

The patients with HIV/AIDS demonstrated a certain acceptance of mobile follow-up services, consistent with the two previous studies (20, 21). Compared with traditional health service mechanisms, mobile information technology has the advantage of offering greater privacy and stronger personalization while providing users with medical services and health services at the time of their choosing, with higher efficiency (22). In this study, the factors influencing the acceptance of mobile information follow-up of patients with HIV/AIDS have been explored by TAM, and the paths of effect explored by SEM, providing more data to inform the use of mobile follow-up services for patients with HIV/AIDS.

Product factors affected their intentions to use mobile information follow-up resources. This was consistent with previous studies on the influencing factors of using similar products. In 2013, for example, a study found that the quality of information available from mobile phone-based HIV interventions for black men significantly affected their intention to use them (23). In 2016, another study emphasized that the website had an attractive layout, colors, fonts, and graphics, inclining users to revisit it (24). The result of this study showed that PFs can indirectly affect BI 2 through PEU or PI, suggesting that patients with HIV/AIDS considered that both convenience and ease of use of mHealth services were affected by the design of the product itself. More concise, accurate, diverse, and easy-to-understand information should therefore be provided for patients with HIV/AIDS. PFs can directly affect BI 3, indicating that the level of user experience of a product may determine whether it is used regularly. When a mobile service product can offer a better user experience, patients will develop a stronger willingness to use it; therefore, when designing a mobile follow-up product, attention should be paid to the users' experience to ensure their long-term commitment to it and should encourage more patients with HIV/AIDS to accept and use the products.

Subjective norms had a direct influence on BI 1, 2, and 3, suggesting that the peer effect was a factor in the acceptance of mobile follow-up services. This result was contrary to a study in 2018 (25) but consistent with another in 2017 (26) which showed that the behavioral intention to use follow-up services gradually increased, from non-use to indispensable use, with patients gradually but finally accepting it and recommending its use to others. The acceptance of users of follow-up services was strengthened by observing others who were using or learning about mHealth application platforms that others recommended. It was, therefore, advocated that peer and authoritative recommendations be increased in the publicity for the service to improve the awareness among patients with HIV/AIDS of mobile follow-up.

Perceived ease of use significantly affected BI, which was consistent with previous research results (27, 28). In this study, we found that PEU directly affects the acceptance of mobile follow-up services by patients with HIV/AIDS, but the impact was not enough to recommend it to others. It only acted indirectly through the PU, being recommended to others only after patients realized the practicality of the product. Previous studies found that currently available online information on HIV and STIs did not meet the needs of men who have sex with men, especially in terms of credibility and practicality (29). The preferences of patients with HIV/AIDS should be taken into consideration to make products cover a wider range of people when designing and implementing mHealth interventions. As a mediating variable between PFs and BI 1, PI performed a mediating role, while, as a mediating variable between PFs and BI 2, PI had a partial mediating role. It suggested that PI was effective in encouraging use by patients with HIV/AIDS. Some studies found that participants were interested in receiving information through Facebook, gay forums, or dating apps and that health interventions should more strongly emphasize interaction with their populations of users (29, 30). It is clear that a diversity of channels through which information and mHealth interventions are provided is very important.

The advantage of this study was that it explored the application of online internet information technology to the management of patients with AIDS with an interdisciplinary perspective. The application of the TAM to explore the receptivity of patients with HIV/AIDS to mobile information follow-up was relatively novel. There are, however, two limitations to this study: (1) the survey was conducted in only one city in Henan Province, China, with a relatively small sample size, though it met the requirements of the TAM, and (2) a self-report questionnaire was used to conduct the survey, so the reliability and validity of the questionnaire may be questionable.

## Conclusion

While other healthcare services for patients with HIV/AIDS were increasing, the acceptance of mHealth was largely determined by how it was perceived by users. In this study, the TAM was used to explore the factors contributing to its acceptance, with those factors following different paths, PFs, PU, PEU, PI, and SNs, all of which can affect BI. Among them, PU had a significant impact on BI, demonstrating that improving the practicality of mHealth services and meeting the needs of patients were crucial. At the same time, convenience and innovation in the experience will be more conducive to the promotion and use of mHealth follow-up services. The views of other patients also had an important impact on their willingness to use the services. Increasing the positive perception of mHealth among the public will incline more patients toward a willingness to receive mHealth follow-up services. This study provides an important reference and rationale for the application of mHealth in follow-up services for patients with HIV/AIDS in China.

## Data availability statement

The original contributions presented in the study are included in the article/Supplementary material, further inquiries can be directed to the corresponding author.

## Ethics statement

The studies involving human participants were reviewed and approved by Zhengzhou University Life Science Ethics Committee. The patients/participants provided their written informed consent to participate in this study.

## Author contributions

CL: methodology and writing—original draft. PW: conceptualization and investigation. CL, MZ, MQ, QC, JM, and HF: data curation and formal analysis. LS: writing—review and editing, supervision and project administration. All authors contributed to the article and approved the submitted version.

## Funding

This study was supported by a scientific research project of the Qingpu District Health Committee (Project No. w2021-44).

## Conflict of interest

The authors declare that the research was conducted in the absence of any commercial or financial relationships that could be construed as a potential conflict of interest.

## Publisher's note

All claims expressed in this article are solely those of the authors and do not necessarily represent those of their affiliated organizations, or those of the publisher, the editors and the reviewers. Any product that may be evaluated in this article, or claim that may be made by its manufacturer, is not guaranteed or endorsed by the publisher.
