# Supplementary material for: Analysis of intention and influencing factors on mobile information follow-up service in HIV/AIDS in a city in China
Source: Front Public Health. 2022 Nov 10;10:997681. doi: 10.3389/fpubh.2022.997681 (PMC9685305; doi:10.3389/fpubh.2022.997681)
Supplement: Supplementary file 1 [file Data_Sheet_1.docx]

Supplementary table 1. Model antecedent variable

| Variable | Variable definition |
| --- | --- |
| Product factors | The information of the official information exchange group expected by users is easy to read, stable, sustainable and updated, and users are used to and like this form to obtain information and communicate. |
| Subjective norm | Users perceive the impact of the views or use of mobile medical AIDS follow-up services of people who are important to them. |
| Perceived ease of use | Users perceive the ease of using mobile medical AIDS follow-up services |
| Perceived usefulness | Users perceive the psychological or physical effects of using mobile medical AIDS follow-up services |
| Perceived innovation | The novelty and advanced sense that users expect to experience after using mobile medical AIDS follow-up service |

Supplementary table 2. Mobile follow-up service acceptance model measurement dimension

| Variable | number | code | question |
| --- | --- | --- | --- |
| Product factors | 4 | A1 | The pictures and texts in the information exchange group are clearly displayed and easy to read |
|  |  | A2 | The information presented by the information exchange group in the mobile phone is constantly updated and stable. |
|  |  | A3 | I am used to reading text in the form of electronic presentation, so it is easy to accept some information from communication groups on my mobile phone. |
|  |  | A4 | I like to use my mobile phone for entertainment and information, so I like to use some chat groups on my mobile phone. |
| Subjective norm | 2 | B1 | If I know that a communication group is already in use among the people I know, I will use it. |
|  |  | B2 | If someone I can trust takes the initiative to recommend a suitable communication group to me, I will use that group. |
| Perceived ease of use | 3 | C1 | I can easily interact with this communication group. |
|  |  | C2 | I can easily operate chat groups like patient groups. |
|  |  | C3 | I can easily find the content related to my health in the group of patients. |
| Perceived usefulness | 3 | C4 | I think using communication groups can help me ease my mood. |
|  |  | C5 | Communication groups enable me to get useful information faster than usual. |
|  |  | C6 | Communication groups are really helpful to my treatment. |
| Perceived Innovation | 2 | C7 | The mobile phone can be carried with me, so joining the mobile communication group allows me to see new information and acquire new knowledge at any time. |
|  |  | C8 | If the communication group is more convenient and advanced than going to the CDC to communicate with the medical staff, I will use the patient group. |
| Behavioral intention | 3 | D1 | I do not reject chat groups such as official exchange groups, and I am willing to use them when the conditions are ripe. |
|  |  | D2 | The communication groups are indispensable to me, and I will use them frequently. |
|  |  | D3 | The communication group is helpful to me, and I would like to recommend it to my patients. |
